# Establishment of a potent weighted risk model for determining the progression of diabetic kidney disease

**DOI:** 10.1186/s12967-023-04245-w

**Published:** 2023-06-12

**Authors:** Tianxiao Zhang, Xiaodan Wang, Yueying Zhang, Ying Yang, Congying Yang, Huiyi Wei, Qingbin Zhao

**Affiliations:** 1 Department of Epidemiology and Biostatistics, School of Public Health, Xi’an Jiaotong University Health Science Center, Xi’an, 710061 Shaanxi China; 2 Department of Geratology, The First Affiliated Hospital of Xi’an Jiaotong University, Xi’an, 710061 Shaanxi China; 3 School of Medicine, Yan’an University, Yan’an, 716000 Shaanxi China; 4 Department of Geratology, The First Affiliated Hospital of Xi’an Jiaotong University, 277 Yanta West Road, Xi’an, 710061 Shaanxi China

**Keywords:** Weighted risk model, Diabetic kidney disease, Progression, Random forest, Dialysis

## Abstract

**Background:**

Diabetic kidney disease (DKD) is a severe complication of diabetes. Currently, no effective measures are available to reduce the risk of DKD progression. This study aimed to establish a weighted risk model to determine DKD progression and provide effective treatment strategies.

**Methods:**

This was a hospital-based, cross-sectional study. A total of 1104 patients with DKD were included in this study. The random forest method was used to develop weighted risk models to assess DKD progression. Receiver operating characteristic curves were used to validate the models and calculate the optimal cutoff values for important risk factors.

**Results:**

We developed potent weighted risk models to evaluate DKD progression. The top six risk factors for DKD progression to chronic kidney disease were hemoglobin, hemoglobin A1c (HbA1c), serum uric acid (SUA), plasma fibrinogen, serum albumin, and neutrophil percentage. The top six risk factors for determining DKD progression to dialysis were hemoglobin, HbA1c, neutrophil percentage, serum albumin, duration of diabetes, and plasma fibrinogen level. Furthermore, the optimal cutoff values of hemoglobin and HbA1c for determining DKD progression were 112 g/L and 7.2%, respectively.

**Conclusion:**

We developed potent weighted risk models for DKD progression that can be employed to formulate precise therapeutic strategies. Monitoring and controlling combined risk factors and prioritizing interventions for key risk factors may help reduce the risk of DKD progression.

**Supplementary Information:**

The online version contains supplementary material available at 10.1186/s12967-023-04245-w.

## Introduction

Diabetic kidney disease (DKD) is a severe microvascular complication of diabetes, an important type of chronic kidney disease (CKD), and a common cause of end-stage renal disease (ESRD). DKD occurs in approximately 30% of patients with type 1 diabetes (T1D) and 40% of those with type 2 diabetes (T2D) [1]. This increase in prevalence coincides with a sharp increase in the global prevalence of diabetes [2]. In the United States, the prevalence of diabetes among adults increased from 9.8% in 1988–1994 to 12.3% in 2011–2012 [3]. Globally, approximately 415 million people were diagnosed with diabetes in 2015. Moreover, the prevalence of diabetes is expected to increase to 642 million by 2040, with a disproportionate increase in low- and middle-income countries [4]. DKD is a major but underrecognized contributor to the global disease burden. The number of deaths due to DKD increased by 94% between 1990 and 2012 [5]. Notably, the increased risks of all-cause and cardiovascular disease mortality in patients with diabetes are associated with DKD [6].

The natural course of DKD includes glomerular hyperfiltration, progressive albuminuria, decreased estimated glomerular filtration rate (eGFR), and eventually ESRD. Diabetes-related metabolic changes can lead to glomerular hypertrophy, sclerosis, tubulointerstitial inflammation, and fibrosis. In terms of pathophysiology, the key metabolic changes that alter renal hemodynamics and promote inflammation and fibrosis in early diabetes include hyperammonemia (a promoter of glomerular hyperfiltration and hyperperfusion) and hyperglycemia [7]. The mechanisms underlying glomerular hyperfiltration in diabetes are not completely understood [5]; however, one plausible mechanism is increased proximal tubular reabsorption of glucose *via* sodium-glucose cotransporter 2, which decreases the distal delivery of solutes, particularly sodium chloride, to the macula densa [8]. The resulting reduction in tubule-glomerular feedback may dilate the afferent arterioles to increase glomerular perfusion, whereas angiotensin II is produced locally in the efferent arterioles in large quantities, leading to vasoconstriction. The overall effect is high intraglomerular pressure and glomerular hyperfiltration [7, 8].

Currently, the known risk factors affecting the progression of DKD include race, hypertension, hyperglycemia, smoking, obesity, toxins, and acute kidney injury, and hyperglycemia and hypertension are highlighted as the two most prominent modifiable risk factors [9]. Clinical guidelines recommend blood glucose control, blood pressure control, smoking cessation, weight loss, and other preventive measures to delay the progression of DKD. Despite the use of current interventions to address many risk factors, there is a large residual risk for DKD progression, suggesting that other risk factors may influence the progression of DKD. As the weights of these risk factors are unknown, it is not known which prevention or treatment strategies should be prioritized. Therefore, extensive innovation is urgently needed to improve the poor kidney outcomes in patients with DKD. To achieve this goal, new treatment strategies must be developed to prevent DKD progression. The aims of this study were to establish weighted risk models for determining DKD progression and derive the optimal cutoff values for key risk factors. Our findings will facilitate the development of strategies for DKD treatment and reduce adverse renal outcomes in patients with DKD.

## Methods

### Study design and participants

This was a hospital-based, cross-sectional study. We randomly selected patients with DKD who were admitted to the First Affiliated Hospital of Xi’an Jiaotong University between January 2018 and December 2020. This study was approved by the Ethics Committee of the First Affiliated Hospital of Xi’an Jiaotong University and was performed in accordance with the Declaration of Helsinki. Written informed consent was obtained from all participants.

A total of 1,104 participants aged 23–96 years were included in the study. Of these participants, 755 (68%) were men, 514 (47%) had an eGFR < 60 mL/min/1.73 m^2^, and 288 (26%) were receiving maintenance dialysis therapy. Adult DKD patients were eligible to participate in this study. The exclusion criteria for patients with DKD were (1) cancer, (2) other secondary nephropathy, (3) other diseases affecting renal function, such as rhabdomyolysis and decompensation of cirrhosis, and (4) autoimmune diseases. As > 95% of the included patients with DKD had T2D, we excluded participants with T1D, gestational diabetes, and other specific types of diabetes. DKD is usually clinically diagnosed based on the presence of albuminuria and/or reduced eGFR in the absence of signs or symptoms of other primary causes of kidney damage [10]. eGFR was estimated using the Chronic Kidney Disease Epidemiology Collaboration equation [11]. A reduced eGFR was defined as < 60 mL/min/1.73 m^2^. Albuminuria was defined as a urinary albumin-to-creatinine ratio ≥ 30 mg/g. The progression of DKD was defined based on two outcomes: progression of DKD to CKD (eGFR < 60 mL/min/1.73 m^2^) and progression of DKD to dialysis (peritoneal dialysis or hemodialysis). The clinical and demographic characteristics of the study participants are summarized in Table 1.

**Table 1:** Clinical and demographical characteristics of the study participants

Variables	eGFR(mL/min/1.73m^2^)		*χ*^*2*^*/t/W*	*P*-value	Dialysis		*χ*^*2*^*/t/W*	*P*-value
	< 60	≥ 60			Yes	No		
	(N = 514)	(N = 590)			(N = 288)	(N = 816)		
Sex (%)								
Male	358(69.6)	397(67.3)	0.60	0.437	205(71.2)	550(67.4)	1.24	0.266
Female	156(30.4)	193(32.7)			83(28.8)	266(32.6)		
Age, years (IQR)	66 (16)	63 (18)	135,932	**0.003**	66 (15)	64 (18)	113,060	0.339
BMI, kg/m^2^ (IQR)	24.7 (4.2)	24.6 (4.4)	151,168	0.930	24.5 (4.0)	24.6 (4.4)	123,570	0.192
Stroke (%)								
Yes	122(23.7)	197(33.3)	12.00	**0.001**	58(20.1)	261(32.0)	13.97	**< 0.001**
No	392(76.3)	393(66.7)			230(79.9)	555(68.0)		
CHD (%)								
Yes	148(28.8)	125(21.1)	8.14	**0.004**	86(29.9)	187(22.9)	5.15	**0.023**
No	366(71.2)	465(78.9)			202(70.1)	629(77.1)		
Diabetes duration, years (IQR)	15 (10)	11 (12)	113,564	**< 0.001**	17 (8)	12 (12)	83,238	**< 0.001**
Smoking status (%)								
Yes	213(41.4)	259(43.9)	0.58	0.446	118(40.9)	354(43.4)	0.41	0.521
No	301(58.6)	331(56.1)			170(59.1)	462(56.6)		
Family history of diabetes (%)								
Yes	165(32.1)	250(42.4)	11.92	**< 0.001**	81(28.1)	334(40.9)	14.34	**< 0.001**
No	349(67.9)	340(57.6)			207(71.9)	482(59.1)		
SBP, mmHg	150.34 ± 23.22	139.31 ± 20.08	−8.39	**< 0.001**	153.13 ± 22.95	141.38 ± 21.23	−7.62	**< 0.001**
DBP, mmHg (IQR)	81 (18)	80 (17)	146,830	0.364	81 (17)	81 (17)	−0.79	0.432
DR (%)								
Yes	356(69.3)	285(48.3)	17.38	**< 0.001**	193(67.0)	448(54.9)	12.33	**< 0.001**
No	158(30.7)	305(51.7)			95(33.0)	368(45.1)		
DF (%)								
Yes	29(5.6)	54(9.2)	3.67	0.055	13(4.5)	68(8.3)	4.02	**0.045**
No	485(94.4)	536(90.8)			275(95.5)	748(91.7)		
Hemoglobin, g/L (IQR)	94.5 (27)	133 (27)	272,494	**< 0.001**	91 (24)	125 (33)	198,100	**< 0.001**
Neutrophil percentage, % (IQR)	72.6 (11.7)	62.4 (12.7)	76,770	**< 0.001**	74.3 (10.1)	64.3 (13.5)	60,740	**< 0.001**
Serum albumin, g/L (IQR)	31.4 (8.7)	38.9 (6.8)	239,666	**< 0.001**	31.3 (8.3)	37.2 (8.7)	167,017	**< 0.001**
Triglyceride, mmol/L (IQR)	1.5 (1.0)	1.4 (1.2)	153,474	0.727	1.4 (0.9)	1.4 (1.2)	125,516	0.085
TC, mmol/L (IQR)	4.0 (1.4)	4.0 (1.4)	152,385	0.887	3.8 (1.3)	4.0 (1.4)	125,382	0.090
Lp(a), mg/L (IQR)	366.5 (398.2)	156.5 (241.8)	88,456	**< 0.001**	392.5 (388.0)	194.5 (313.0)	76,886	**< 0.001**
LDL-C, mmol/L (IQR)	2.2 (1.1)	2.3 (1.1)	158,023	0.226	2.2 (1.1)	2.3 (1.2)	125,017	0.106
HDL-C, mmol/L (IQR)	0.96 (0.39)	0.92 (0.33)	149,801	0.729	0.92 (0.39)	0.94 (0.34)	125,204	0.098
SUA, µmol/L (IQR)	386 (149)	317 (119)	98,993	**< 0.001**	370 (160)	336 (128)	99,506	**< 0.001**
HbA1c, % (IQR)	6.5 (1.8)	8.6 (3.1)	237,037	**< 0.001**	6.3 (1.5)	8.0 (3.0)	180,785	**< 0.001**
Plasma fibrinogen, g/L (IQR)	4.4 (1.6)	3.3 (1.4)	76,625	**< 0.001**	4.4 (1.5)	3.5 (1.7)	78,807	**< 0.001**

Continuous variables that passed the normality check are expressed as mean ± standard deviation, and t-tests were used to compare the differences between the two groups. Variables which failed to pass the normality check are presented as median (IQR) and the Mann-Whitney U test was performed to compare the differences between the two groups

Significant *P* values are indicated in Bold

*BMI* body mass index, *CHD* coronary heart disease, *DBP* diastolic blood pressure, *DF* diabetic foot, *DR* diabetic retinopathy, *eGFR* estimated glomerular filtration rate, *HbA1c* hemoglobin A1c, *HDL-C* high-density lipoprotein cholesterol, *IQR* interquartile range, *LDL-C* low-density lipoprotein cholesterol, *Lp(a)* lipoprotein(a), *SBP* systolic blood pressure, *SUA* serum urea acid, *TC* total cholesterol

### Clinical indicator measurements

A total of 23 variables were included as candidate risk factors for DKD progression based on previous literature and expert opinions. These 23 variables were classified into three categories. The demographic characteristics of the study participants were sex, age, body mass index (BMI), smoking status, and family history of diabetes. The clinical information included duration of diabetes, history of coronary heart disease (CHD), stroke, systolic blood pressure (SBP), diastolic blood pressure (DBP), diabetic retinopathy (DR), and diabetic foot (DF) status. In addition, 11 blood biochemical indicators that may contribute to the risk assessment of DKD progression were included in the assessment. These biochemical indicators included whole blood hemoglobin and hemoglobin A1c (HbA1c) levels, neutrophil percentage, serum albumin, uric acid (SUA), total cholesterol (TC), triglycerides, low-density lipoprotein cholesterol (LDL-C), high-density lipoprotein cholesterol (HDL-C), lipoprotein(a) [Lp(a)], and plasma fibrinogen levels.

Body mass index (BMI) was calculated as weight in kilograms divided by the square of height in meters measured at admission. Blood pressure was measured using a mercury sphygmomanometer in a quiet state upon admission. A history of CHD was confirmed by medical records or defined as a history of angina pectoris or myocardial infarction, positive cardiac stress test results, or pathological signs on coronary angiography [12]. A history of stroke was confirmed by the presence of any neurological deficiency, with or without sequelae [12]. Each participant was examined by a professional ophthalmologist using wide-area fundus photography, optical coherence tomography, and fundus fluorescein angiography to determine the presence of DR. DF was confirmed in patients with diabetes who developed wounds secondary to neuropathy, with or without biomechanical abnormalities, peripheral arterial disease, or both [13].

Venous blood was collected from all participants after an overnight fast of at least 10 h for biochemical tests. SUA, serum creatinine, and albumin levels were determined using an automatic biological analyzer (HITACHI, LABOSPECT008). The percentages of neutrophils and hemoglobin in whole blood were measured using an automatic analyzer (MINDRAY 6800 plus). HbA1c levels were determined using high-performance liquid chromatography. Plasma fibrinogen levels were determined using a class coagulation method. Serum levels of triglycerides and TC were assessed using an enzymatic colorimetric method, and HDL-C and LDL-C levels were assessed using the direct method. Serum Lp(a) levels were determined using an immunoturbidimetric method. The urinary creatinine and albumin levels were assessed using a rate-nephelometric assay.

### Statistical analysis

**Fig. 1:**
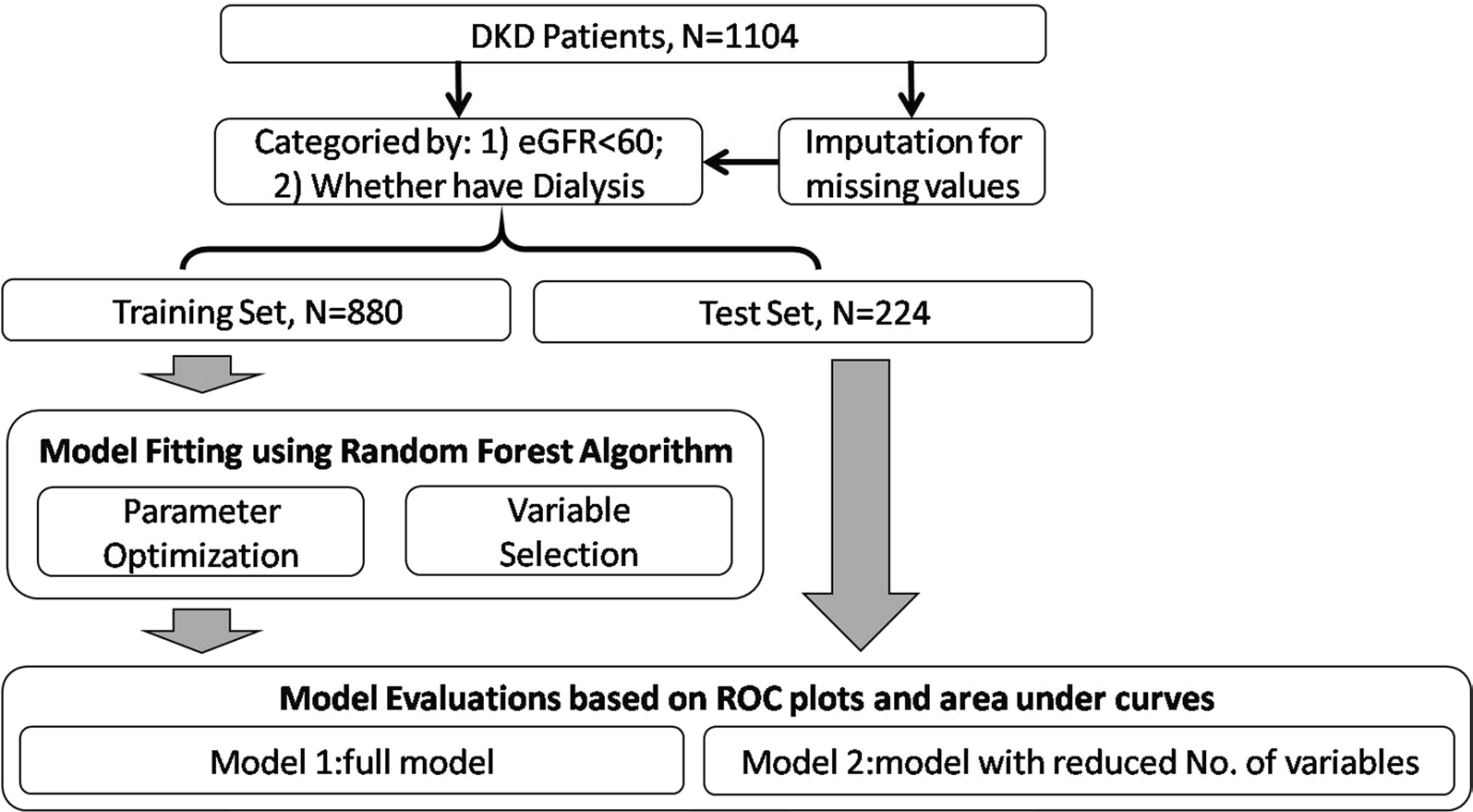
Study design and analysis framework

Machine learning models using random forest (RF) algorithms were fitted to evaluate the performance of multiple clinical and demographic variables as potential risk indicators of DKD progression. Two DKD-related adverse outcomes were used to fit the models. A total of 23 clinical and demographic variables were selected as features of the machine learning models. A flowchart of the analysis is provided in Fig. 1.

The data from patients with DKD were randomly split into training and test sets (at a ratio of 8:2). For both outcomes, the RF models were constructed based on full and reduced feature sets to remove the potential effects of overfitting. The number of variables randomly sampled as candidates at each split (parameter “mtry”) were optimized based on the mean error rate of the RF model for DKD patients in the training set (Additional file 1: Figure S1 and S3). The number of features retained in the reduced models was determined based on out-of-bag errors in the RF model in patients with DKD (Additional file 1: Figure S2 and S4). For the model using eGFR level as the outcome, six features were selected in the reduced model. For the model using dialysis as the outcome, 12 features were selected from the reduced model. The features used in the full set and reduced models are summarized in Additional file 1: Table S1. The importance of these features as indicators of DKD progression was measured based on the mean decrease in accuracy of the RF models. The performance of the models was depicted based on the receiver operating characteristic curve and measured using the area under the curve (AUC). Youden’s index was also computed for specific indicators to determine the optimal cutoff values.

Normality check was performed using Shapiro-Wilk’s test. Continuous variables are expressed as mean ± standard deviation, and t-tests were performed to compare the differences between the two groups. Variables which failed to pass the normality check are presented as median with interquartile range (IQR) and the Mann-Whitney U test was performed to compare the differences between the two groups. Categorical variables are expressed as percentages, and the chi-square test was conducted to compare differences between the two groups. Statistical significance was set at *P* < 0.05. Statistical analyses and model fitting were performed using R software and its relevant packages (version 4.2.2).

## Results

### Clinical and demographic characteristics of the participants

A total of 1,104 patients with DKD were recruited for this study. The clinical and demographic characteristics of the study participants are summarized in Table 1. In general, the distributions of the 23 variables differed between the outcome groups defined in this study. For the outcomes classified based on eGFR level, nine of the 23 features, including sex, BMI, smoking status, DBP, DF, triglyceride, TC, LDL-C, and HDL-C, were not significantly different between the two groups. For the outcome groups defined by dialysis, nine of the 23 features, including sex, age, BMI, smoking status, DBP, triglyceride, TC, LDL-C, and HDL-C, were not significantly different.

### Model performance for the two outcomes

**Fig. 2:**
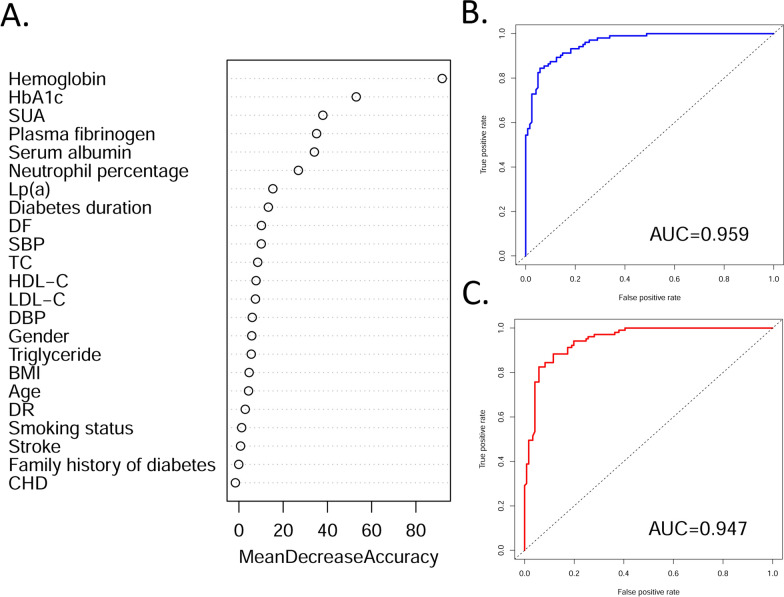
Feature importance and model performance for DKD patients classified by eGFR. **A** Feature importance of the model; **B** ROC curve and AUC value for the random forest model constructed with full features (n = 23); **C** ROC curve and AUC value for the random forest model constructed with selected features (n = 6) * AUC* area under the curve, *BMI* body mass index, *CHD* coronary heart disease, *DBP* diastolic blood pressure, *DF* diabetic foot, *DKD* diabetic kidney disease, *DR* diabetic retinopathy, *eGFR* estimated glomerular filtration rate, *HbA1c* hemoglobin A1c, *HDL-C* high-density lipoprotein cholesterol, *LDL-C* low-density lipoprotein cholesterol, *Lp(a)* lipoprotein(a), *ROC* receiver operating characteristic, *SBP* systolic blood pressure, *SUA* serum urea acid, *TC* total cholesterol

**Fig. 3:**
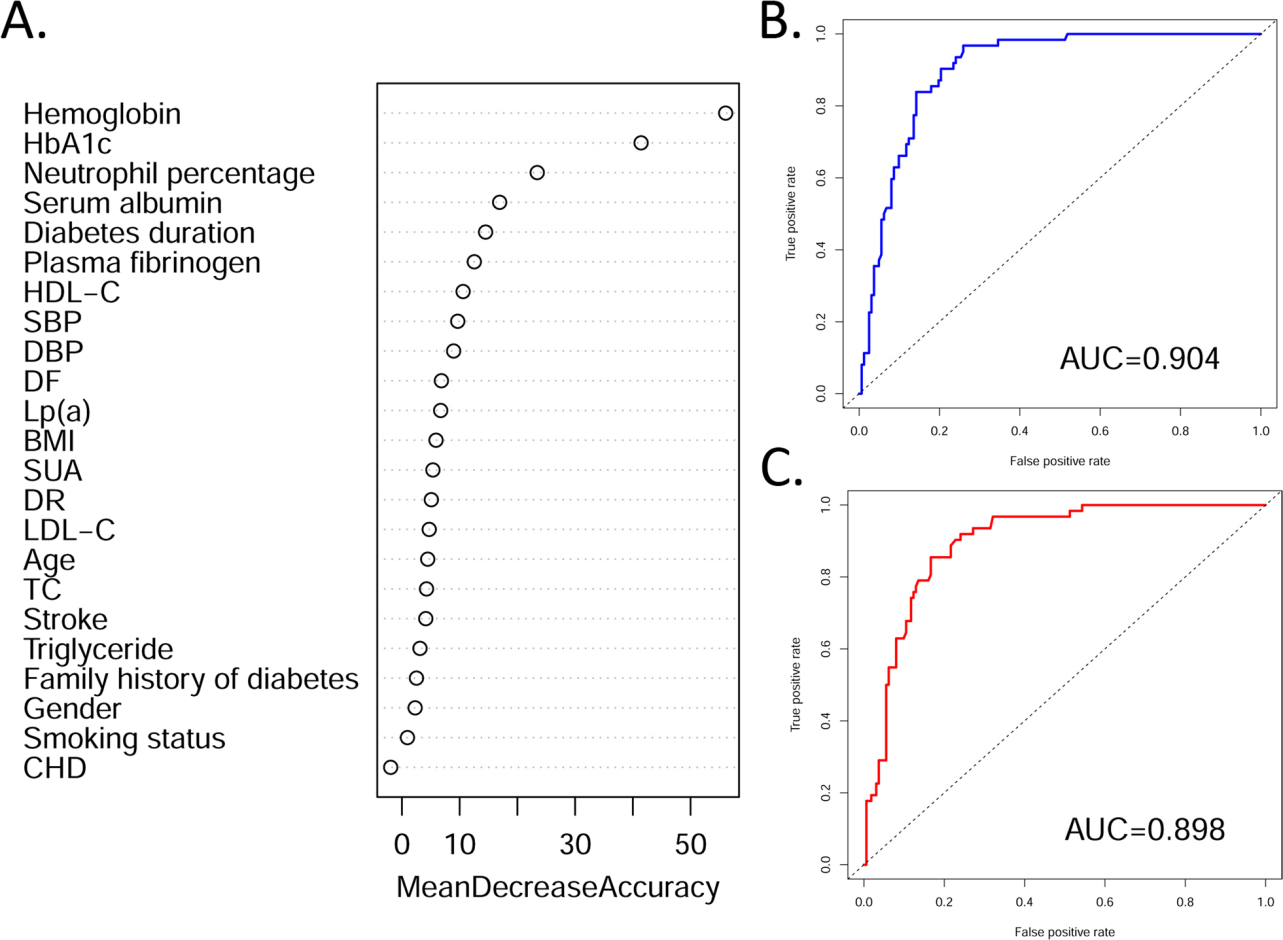
Feature importance and model performance for DKD patients classified by dialysis. **A** Feature importance of the model; **B** ROC curve and AUC value for the random forest model constructed with full features (n = 23); **C** ROC curve and AUC value for the random forest model constructed with selected features (n = 12) * AUC* area under the curve, *BMI* body mass index, *CHD* coronary heart disease, *DBP* diastolic blood pressure, *DF* diabetic foot, *DKD* diabetic kidney disease, *DR* diabetic retinopathy, *HbA1c* hemoglobin A1c, *HDL-C* high-density lipoprotein cholesterol, *LDL-C* low-density lipoprotein cholesterol, *Lp(a)* lipoprotein(a), *ROC* receiver operating characteristic, *SBP* systolic blood pressure, *SUA* serum urea acid, *TC* total cholesterol

The RF models were fitted to DKD patients with different outcomes. For the outcomes classified based on reduced eGFR, the six most important features were hemoglobin, HbA1c, SUA, plasma fibrinogen, serum albumin, and neutrophil percentage (Fig. 2A). The AUC [95% confidence interval (CI)] of the RF model with all variables (n = 23) for reduced eGFR in the test set was 0.959 (0.937–0.981) (Additional file 1: Table S2 and Fig. 2B). Although this measurement slightly decreased in the reduced feature set model (n = 6), with an AUC (95% CI) of 0.947 (0.921–0.974) (Fig. 2C), the six-variable RF model was sufficiently effective at evaluating the progression of DKD to CKD. Similar patterns were observed for the dialysis outcome, despite lower AUC measurements than those of the model for eGFR levels (Fig. 3). The 12 most important features were hemoglobin, HbA1c, neutrophil percentage, serum albumin, duration of diabetes, plasma fibrinogen, HDL-C, SBP, DBP, DF, Lp(a), and BMI (Fig. 3A). The AUC (95% CI) of the RF model for dialysis in the test set was 0.904 (0.865–0.943) (Fig. 3B) for the full feature set (n = 23). For the reduced feature set model (n = 12), this measurement decreased to 0.898 (0.857–0.940) (Fig. 3C); however, the model was still powerful enough for evaluating DKD progression to dialysis.

### Optimal cutoff values for the single-indicator model with hemoglobin or HbA1c

**Fig. 4:**
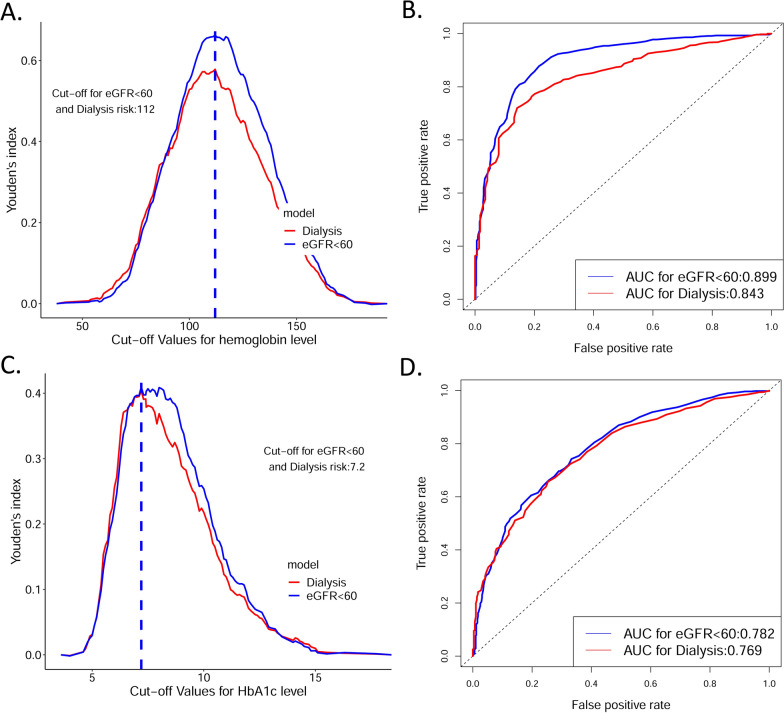
Cutoff values and model performance of the single important feature. **A** Youden’s index and cutoff values for eGFR < 60 mL/min/1.73 m^2^ and dialysis using hemoglobin level as a single feature; **B** ROC curves and AUC values for eGFR < 60 mL/min/1.73 m^2^ and dialysis using hemoglobin level as a single feature; **C** Youden’s index and cutoff values for eGFR < 60 mL/min/1.73 m^2^ and dialysis using HbA1c level as a single feature; **D** ROC curves and AUC values for eGFR < 60 mL/min/1.73 m^2^ and dialysis using Hb A1c level as a single feature * AUC* area under the curve, *eGFR* estimated glomerular filtration rate, *HbA1c* hemoglobin A1c, *ROC* receiver operating characteristic

The performance of the single-indicator models was examined to explore their potential at predicting DKD progression. The two most important features, hemoglobin and HbA1c levels, were examined. The optimal cutoff values (the highest Youden’s index) of hemoglobin and HbA1c were the same for the two types of outcomes, 112 g/L and 7.2%, respectively (Fig. 4A, C). Hemoglobin, as a single indicator of DKD progression, achieved an AUC (95% CI) of 0.899 (0.880–0.917) for the outcome defined by reduced eGFR and 0.843 (0.818–0.868) for the outcome of dialysis (Fig. 4B). In contrast, HbA1c as a single feature achieved an AUC (95% CI) of 0.782 (0.755–0.808) for the outcome defined by reduced eGFR and 0.769 (0.739-0.800) for the outcome of dialysis (Fig. 4D). The sensitivity and specificity of the single-marker models using Youden’s index cutoff are summarized in Additional file 1: Table S3.

## Discussion

To the best of our knowledge, this is the first study to use RF models to investigate the weighted rankings of multiple risk factors that affect DKD progression. Based on our findings, many risk factors affect the progression of DKD, and weighted relationships exist among different risk factors. Herein, a powerful weighted risk model was established to evaluate DKD progression to CKD. The top six risk features in the model were hemoglobin, HbA1c, SUA, plasma fibrinogen, serum albumin, and neutrophil percentage. An effective weighted risk model was also developed to assess DKD progression to dialysis; the top six risk factors in the model were hemoglobin, HbA1c, neutrophil percentage, serum albumin, duration of diabetes, and plasma fibrinogen. The optimal cutoff values of hemoglobin and HbA1c for determining DKD progression were 112 g/L and 7.2%, respectively. Overall, our findings highlight the weighted relationship between the clinical features that influence the progression of DKD, which may provide precise intervention strategies for delaying the progression of DKD.

Decreased hemoglobin levels were demonstrated to be an important clinical feature in patients with progressive DKD, ranking first in terms of weighted risk. In clinical practice, patients with DKD and significantly decreased renal function often have significant anemia, whereas patients with normal or mildly decreased renal function have no obvious anemia. A recent study reported a higher prevalence of anemia in T2D patients with non-dialysis CKD, and anemia prevalence increased as the CKD worsened [14]. The known mechanisms of anemia in DKD include malnutrition due to insufficient erythropoietic materials (such as iron, folic acid, and vitamin B12), blood loss, chronic inflammation, and dyserythropoiesis caused by decreased secretion or defective action of erythropoietin in the kidney [15]. Furthermore, for the first time, we demonstrated that a hemoglobin level of < 112 g/L was the optimal cutoff value for evaluating eGFR decline or dialysis. Therefore, our results suggest that hemoglobin level is an important and easily obtained blood biochemical marker for assessing the adverse outcomes of DKD, and the correction of anemia for different etiologies may delay the progression of DKD.

Hyperglycemia is an important controllable risk factor for DKD [16]. Intensive glycemic control to achieve near-normoglycemia has been shown in large prospective randomized studies to delay the onset and progression of albuminuria and reduce eGFR in patients with T1D [17] and T2D [18]. However, decreased HbA1c level was identified as an important clinical feature in patients with progressive DKD; this feature was ranked second in our risk model. In addition, based on our calculation, the optimal cutoff value of HbA1c to assess progressive DKD was 7.2%. The 2022 American Diabetes Association (ADA) guidelines recommend that an HbA1c goal of < 7% for many non-pregnant adults without significant hypoglycemia is appropriate; however, the goal of HbA1c in patients with DKD should be patient-centered and individualized, and the adverse risk of hypoglycemia in elderly people and patients with renal insufficiency should be considered [19]. The mechanisms by which hyperglycemia promotes DKD progression include accumulation of advanced glycation end products, epigenetic changes, and oxidative stress [20]. However, the HbA1c level in progressive DKD patients was close to 7%, which could not accurately reflect blood glucose control because HbA1c levels are affected by hemoglobin, and progressive DKD patients often have anemia. Therefore, HbA1c levels would be underestimated, and the illusion of good blood glucose control would be obtained. Therefore, HbA1c is not an effective blood glucose monitoring indicator for patients with progressive DKD. In fact, the time throughout the day when blood glucose is within the normal range, obtained using a continuous glucose monitoring system, may more accurately guide blood glucose management in patients with DKD. Although both the ADA and Kidney Disease: Improving Global Outcomes (KDIGO) guidelines focus on HbA1c as the primary tool for assessing long-term glycemic control, both acknowledge limitations in its accuracy as an indirect metric of glycemic status, particularly in advanced CKD and kidney failure treated with dialysis, and the inability of HbA1c to adequately capture glycemic variability and hypoglycemic events [21].

Higher SUA levels were associated with an increased risk of DKD progression [22]. In this study, elevated SUA levels were found to be a significant biochemical feature in patients with progressive DKD and ranked third in the weight of multiple risk factors. A meta-analysis revealed that hyperuricemia is associated with worsening eGFR, albuminuria, CKD, and kidney failure [23]. Inflammation [24], insulin resistance [25], intrarenal hemodynamic dysfunction [26], vascular, glomerular, and tubular damage [25], and nephron mass loss [27] have been revealed to explain the etiological relationship between elevated SUA levels in T2D and vascular disease, and how elevated SUA accelerates the progression of DKD. Therefore, our findings suggest that lowering SUA levels may delay DKD progression.

Based on our findings, elevated plasma fibrinogen is an important biochemical feature of progressive DKD, ranking fourth and sixth in the weight of multiple risk features in DKD patients with reduced eGFR or dialysis, respectively. The mean plasma fibrinogen level in these patients was 4.6 g/L, which is significantly higher than the normal high limit of 4 g/L. Fibrinogen is a soluble glycoprotein synthesized by the liver and plays a vital role in coagulation and inflammation [28]. DKD is an inflammatory disease [29]. Elevated plasma fibrinogen levels are associated with ESRD in patients with T2D [30]. Therefore, the plasma fibrinogen levels in patients with DKD should be regularly monitored in clinical practice. Early detection and reduction of elevated plasma fibrinogen levels may delay DKD progression.

Decreased serum albumin level was identified as a key risk factor for progressive DKD. Serum albumin maintains most of the intravascular colloidal osmotic pressure and plays a crucial role as a carrier protein and antioxidant [31]. A prospective study revealed that decreased serum albumin level is a risk factor for all-cause mortality in patients with CKD [32]. Another study revealed that decreased serum albumin levels may be an independent risk indicator of DKD progression in patients with T2D [33]. Possible mechanisms for the serum albumin decline in patients with DKD include damaged glomeruli leaching large amounts of albumin; reduced nutrient (protein and calories) intake, which might be due to anorexia secondary to uremic toxins, slowing of gastric emptying; systemic inflammation; and comorbidities [34].

Infection is another important risk factor for renal deterioration and death in patients with CKD, especially those undergoing dialysis [35]. Neutrophils are the most abundant white blood cells in circulation. Patients with congenital neutrophil deficiencies suffer from severe infections that are often fatal, underscoring the importance of these cells in immune defense. Neutrophils serve not only as professional killers, but also as immune system guides during infections and inflammatory diseases [36]. This study revealed that elevated blood neutrophil percentage is an important biochemical feature of progressive DKD, suggesting that infection contributes to adverse renal outcomes in patients with DKD. Patients with DKD may develop multisystem bacterial infections, such as those of the digestive, respiratory, and urinary tracts, owing to poor blood glucose control and decreased systemic immunity. The mechanisms by which infections promote renal function deterioration in DKD involve inflammatory responses and immune dysregulation [35]. A growing body of evidence suggests that chronic inflammatory damage to the kidney underlies many of the structural and functional changes in DKD. Activated inflammatory molecules and pathways, including cytokines, chemokines, innate immune cells, and adhesion molecules, mediate the initiation and progression of DKD [37]. Thus, preventing and treating underlying infections, reducing elevated inflammatory responses, and improving autoimmunity may delay the progression of DKD.

Hypertension is closely associated with DKD [38]. The mechanisms underlying hypertension in patients with CKD include increased salt and volume retention, upregulation of the renin-angiotensin system, endothelial dysfunction, and increased sympathetic activity [39]. In this study, SBP was found to be more important than DBP in affecting the progression of DKD; that is, the increase in SBP was more obvious in patients with DKD with an eGFR decline or those on dialysis, suggesting that effective control of SBP may delay the progression of DKD. However, in clinical practice, the goal of blood pressure control in patients with DKD is to improve with an increase in evidence-based data. The Association of British Clinical Diabetologists and the Renal Association UK guidelines recommend that for T2D patients with normal renal function and microalbuminuria or CKD stages 1–3, blood pressure control of < 130/80 mmHg should be achieved [40]. For patients with T2D and CKD stages 4–5 (non-dialysis), a higher target of < 140/90 mmHg may be appropriate for adults > 65 years, whereas for patients with albuminuria, the blood pressure control target is < 130/80 mmHg [40]. For patients with T2D with CKD stage 5 (on dialysis), blood pressure control of < 140/90 mmHg should be achieved [40]. Our study revealed that the average SBP in DKD patients with decreased eGFR or those on dialysis was approximately 150 mmHg, whereas the average SBP in DKD patients with mild or no dialysis was approximately 140 mmHg, suggesting that an SBP < 140 mmHg may be appropriate for delaying DKD progression.

This study had several limitations. First, cross-sectional data were used, which did not allow us to determine the temporal link between the selected risk factors and DKD progression. Further prospective studies are required to confirm this association. Second, we did not obtain information on long-term blood glucose control indicators, such as the time to blood glucose normalization, or information on medications, such as angiotensin-converting enzyme inhibitors/angiotensin receptor antagonists, sodium-glucose transporter 2 inhibitors, and glucagon-like peptide 1 receptor agonists, which may have reduced our ability to explore other risks or protective factors. Finally, as this was a retrospective study, the cause-and-effect relationships could not be established.

## Conclusions

In summary, we developed powerful weighted risk models to assess DKD progression. These findings provide an important basis for the optimization of therapeutic strategies for DKD by monitoring and controlling combined risk factors and prioritizing interventions for key risk factors, which may help reduce the risk of DKD progression. Further prospective studies are required to clarify whether combined interventions with important risk factors can reduce the residual risk of DKD progression.

## Supplementary Information

**Additional file 1:** Detail information on parameter selection and optimization, variable selection and model performance measured by multiple indicators.

## Data Availability

The datasets generated and/or analyzed during the current study are not publicly available due to local legal requirements but are available from the corresponding author on reasonable request.
