# Supplementary material for: Establishment of a potent weighted risk model for determining the progression of diabetic kidney disease
Source: J Transl Med. 2023 Jun 12;21:381. doi: 10.1186/s12967-023-04245-w (PMC10259039; doi:10.1186/s12967-023-04245-w)
Supplement: Supplementary file 1 — Additional file 1: Detail information on parameter selection and optimization, variable selection and model performance measured by multiple indicators. [file 12967_2023_4245_MOESM1_ESM.docx]

**Additional file Tables**

**Table S1**  Features kept in reduced models

| **Full Feature Set Model** | **Reduced Model 1** | **Reduced Model 2** |
| --- | --- | --- |
| Gender | Hemoglobin | Hemoglobin |
| Age | HbA1c | HbA1c |
| BMI | SUA | Neutrophil percentage |
| Stroke | Plasma fibrinogen | Serum albumin |
| CHD | Serum albumin | Diabetes duration |
| Diabetes duration | Neutrophil percentage | Plasma fibrinogen |
| Smoking Status |  | HDL-C |
| Family history of diabetes |  | SBP |
| SBP |  | DBP |
| DBP |  | DF |
| DR |  | Lp(a) |
| DF |  | BMI |
| Hemoglobin |  |  |
| Neutrophil percentage |  |  |
| Serum albumin |  |  |
| Triglyceride |  |  |
| TC |  |  |
| Lp(a) |  |  |
| LDL-C |  |  |
| HDL-C |  |  |
| SUA |  |  |
| HbA1c |  |  |
| Plasma fibrinogen |  |  |

BMI: body mass index; CHD: coronary heart disease; DBP: diastolic blood pressure; DR: diabetic retinopathy; DF: diabetic foot; HbA1c: hemoglobin A1c; HDL-C: high-density lipoprotein cholesterol; LDL-C: low-density lipoprotein cholesterol; Lp(a): lipoprotein(a); SBP: systolic blood pressure; SUA: serum uric acid; TC: total cholesterol.

Model 1: the random forest model using eGFR level as outcome. eGFR: estimated glomerular filtration rate.

Model 2: the random forest model using dialysis status as outcome.

**Table S2** AUCs and the 95% confidence intervals for different random forest models.

| **Model** | **AUC (95% CI)** |
| --- | --- |
| DKD patients classified by eGFR |  |
| Full Features | 0.959 (0.937-0.981) |
| Selected Features | 0.947 ( 0.921-0.974) |
| Single Feature: Hemoglobin | 0.899 ( 0.880-0.917) |
| Single Feature: HbA1c | 0.782 ( 0.755-0.808) |
| DKD patients classified by dialysis |  |
| Full Features | 0.904 (0.865-0.943) |
| Selected Features | 0.898 (0.857-0.940) |
| Single Feature: Hemoglobin | 0.843 ( 0.818-0.868) |
| Single Feature: HbA1c | 0.769 ( 0.739-0.800) |

AUC: area under the curve; CI: confidence interval; DKD: diabetic kidney disease; eGFR: estimated glomerular filtration rate; HbA1c: hemoglobin A1c.

**Table S3** Sensitivity and specificity for single marker models using Youden's index cutoff.

| **Model** | **Sensitivity (95% CI)** | **Specificity (95% CI)** |
| --- | --- | --- |
| Hemoglobin |  |  |
| eGFR | 0.78 (0.74-0.83) | 0.88 (0.83-0.92) |
| Dialysis | 0.86 (0.80-0.90) | 0.72 (0.62-0.76) |
| HbA1c |  |  |
| eGFR | 0.67 (0.74-0.83) | 0.74 (0.68-0.78) |
| Dialysis | 0.75 (0.68-0.79) | 0.66 (0.58-0.71) |

CI: confidence interval; eGFR: estimated glomerular filtration rate; HbA1c: hemoglobin A1c.

**Additional file Figures**


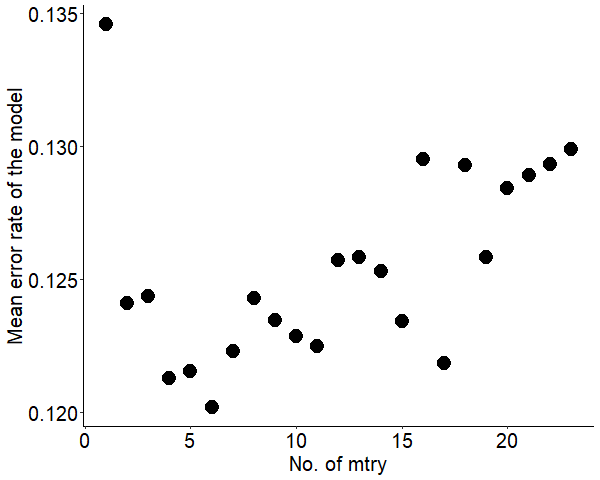


**Figure S1** Mean error rate of the random forest model for DKD patients classified by eGFR with different values of number of mtry.

DKD: diabetic kidney disease; eGFR: estimated glomerular filtration rate.


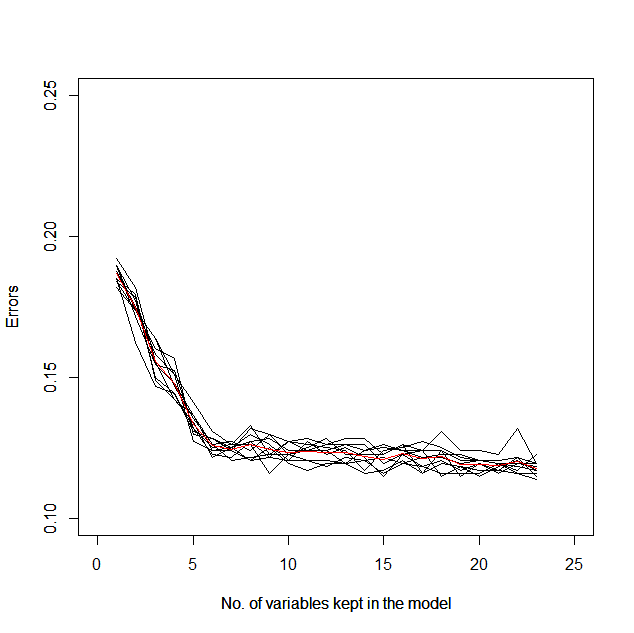


**Figure S2** Out-of-bag errors of the random forest model for DKD patients classified by eGFR with different number of variables kept. The process was repeated by 10 times and the average values were indicated in red.

DKD: diabetic kidney disease; eGFR: estimated glomerular filtration rate.


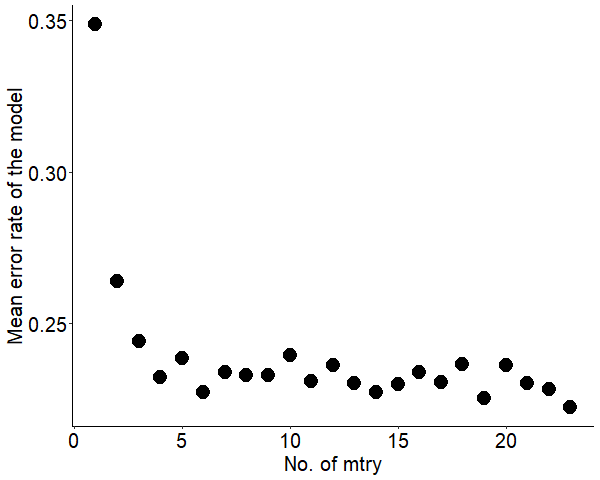


**Figure S3** Mean error rate of the random forest model for DKD patients classified by dialysis with different values of number of mtry.

DKD: diabetic kidney disease.


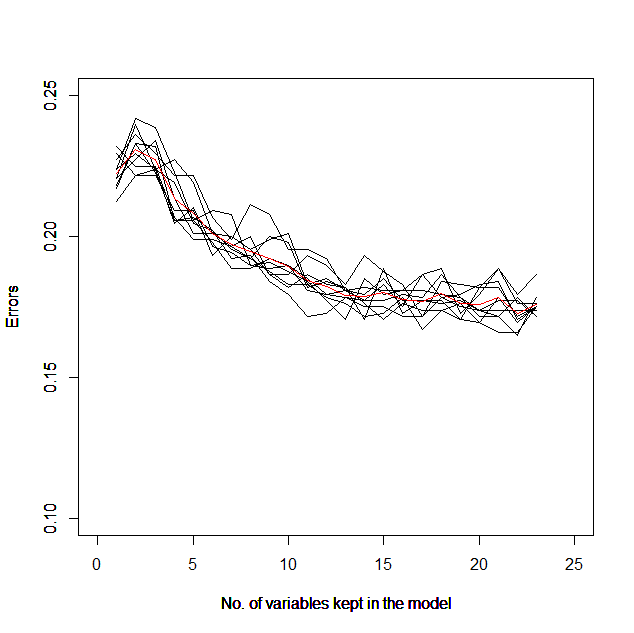


**Figure S4** Out-of-bag errors of the random forest model for DKD patients classified by dialysis with different number of variables kept. The process was repeated by 10 times and the average values were indicated in red.

DKD: diabetic kidney disease.
